# Deep learning classification for macrophage subtypes through cell migratory pattern analysis

**DOI:** 10.3389/fcell.2024.1259037

**Published:** 2024-02-07

**Authors:** Manasa Kesapragada, Yao-Hui Sun, Ksenia Zlobina, Cynthia Recendez, Daniel Fregoso, Hsin-Ya Yang, Elham Aslankoohi, Rivkah Isseroff, Marco Rolandi, Min Zhao, Marcella Gomez

**Affiliations:** ^1^ Department of Applied Mathematics, Baskin School of Engineering, University of California, Santa Cruz, Santa Cruz, CA, United States; ^2^ Department of Ophthalmology and Vision Science, School of Medicine, University of California, Davis, Sacramento, CA, United States; ^3^ Department of Dermatology, School of Medicine, UC Davis, Sacramento, CA, United States; ^4^ Department of Electrical and Computer Engineering, Baskin School of Engineering, University of California, Santa Cruz, Santa Cruz, CA, United States

**Keywords:** correlation between cell shape and trajectories, macrophage polarization, classification of macrophage subtypes using migration patterns, deep learning for classification of macrophage subtypes, analysis of macrophage trajectory patterns

## Abstract

Macrophages can exhibit pro-inflammatory or pro-reparatory functions, contingent upon their specific activation state. This dynamic behavior empowers macrophages to engage in immune reactions and contribute to tissue homeostasis. Understanding the intricate interplay between macrophage motility and activation status provides valuable insights into the complex mechanisms that govern their diverse functions. In a recent study, we developed a classification method based on morphology, which demonstrated that movement characteristics, including speed and displacement, can serve as distinguishing factors for macrophage subtypes. In this study, we develop a deep learning model to explore the potential of classifying macrophage subtypes based solely on raw trajectory patterns. The classification model relies on the time series of x-y coordinates, as well as the distance traveled and net displacement. We begin by investigating the migratory patterns of macrophages to gain a deeper understanding of their behavior. Although this analysis does not directly inform the deep learning model, it serves to highlight the intricate and distinct dynamics exhibited by different macrophage subtypes, which cannot be easily captured by a finite set of motility metrics. Our study uses cell trajectories to classify three macrophage subtypes: M0, M1, and M2. This advancement holds promising implications for the future, as it suggests the possibility of identifying macrophage subtypes without relying on shape analysis. Consequently, it could potentially eliminate the necessity for high-quality imaging techniques and provide more robust methods for analyzing inherently blurry images.

## 1 Introduction

Macrophages are involved in the body’s immune responses and tissue homeostasis. They play a critical role in infectious diseases (Atri et al., 2018), cancer progression (Chanmee et al., 2014), autoimmunity (Funes et al., 2018), wound healing (Krzyszczyk et al., 2018), and many other diseases (Murray et al., 2014; Wynn and Vannella, 2016). Two main subsets of activated macrophages with different functional phenotypes, M1 (classically activated, pro-inflammatory) and M2 (alternatively activated, anti-inflammatory), have been identified (Martinez and Gordon, 2014; Murray et al., 2014). In tumor progression, M1 and M2 macrophages assume distinct roles. Specifically, the M2 subtype, M2d, demonstrates pro-neoplastic characteristics, while M1-like macrophages exert anti-tumor effects (Sica et al., 2008). Recent studies have shown that besides M1 and M2 types, a continuum of macrophage subtypes exists (Mosser and Edwards, 2008; Martinez and Gordon, 2014).

The study of macrophage subtypes plays a crucial role in identifying strategies for disease control (Kotwal and Chien, 2017; Chatterjee et al., 2021; Du et al., 2021; Kuntzel and Bagnard, 2022). Consequently, developing effective methods for detecting macrophage subtypes *in vitro* is essential.

The conventional method to identify M1 and M2 subtypes involves analyzing multiple cell surface markers, transcription factors, and cytokine profiles, which can be time-consuming and resource-intensive. Furthermore, uncertainty remains about how to identify macrophage subtypes confidently. This is in part due to the existing continuum of states. Recent studies of macrophage cultures led researchers to hypothesize that cell morphology could indicate macrophage activation status (Rostam et al., 2017).

Previous research on the classification of macrophage subtypes using machine learning has been based on fluorescent dyes and cell shape parameters (Mcwhorter et al., 2013; Rostam et al., 2017; Pavillon et al., 2018). More recently, it was suggested that motility parameters like cell speed could be used to classify macrophage subtypes (Kesapragada et al., 2024). These publications show that, although a continuum of phenotypes exists, there indeed are three primary shape modes associated with three distinct phenotypes, respectively (inclusive of the so-called “naïve” macrophages). Furthermore, it was shown that these shape modes are closely linked with predetermined cell motility metrics.

This research aims to gain a deeper understanding of macrophages’ migratory patterns and explore the potential of classifying macrophage subtypes based on raw trajectory patterns without relying on cell shape analysis. This could provide more robust methods to analyze blurry images.

We suggest that a holistic use of cell motility information, i.e., a time series of cell coordinates, could enhance the differentiation of macrophage subtypes. We develop a deep learning model that uses cell position over time as input and demonstrate that our model effectively distinguishes between M1 and M2 macrophage phenotypes. This classification method could potentially be used to understand the continuum of states further.

## 2 Macrophages migratory pattern analysis

In this study, we leverage labelled data published in our previous work (Kesapragada et al., 2024)). In brief, bone marrow-derived macrophages (BMDMs) were isolated and cultured, resulting in an M0 macrophage culture. M0 macrophages were further activated into either M1 (with LPS) or M2 (with IL-4) (see Methods) in correspondence with existing protocols (Zhang et al., 2008; Ying et al., 2013). Time-lapse recording of cell images, segmentation, and tracking of cell trajectories was performed in correspondence with Magnusson et al. (2015). This global track-linking algorithm links cell outlines generated by a segmentation algorithm into tracks. Tracks are incrementally added to the image sequence using information from the complete image sequence in every linking decision.

Overall, we obtained three videos of single cells and three videos of cell cultures.

Single-cell video sequences were captured for one M0 cell, one M1 cell, and one M2 cell, respectively. Each single-cell video comprises 240 images, with a frame interval of 1 min, resulting in a total duration of 240 min.

Cell-culture video sequences were obtained from a non-activated M0 cell culture, an M1-activated cell culture, and an M2-activated cell culture, respectively. Each cell culture video comprises 37 phase contrast frames, each frame captured at a 5-minute interval, spanning a total duration of 180 min.

### 2.1 Single-cell macrophages images

**FIGURE 1:**
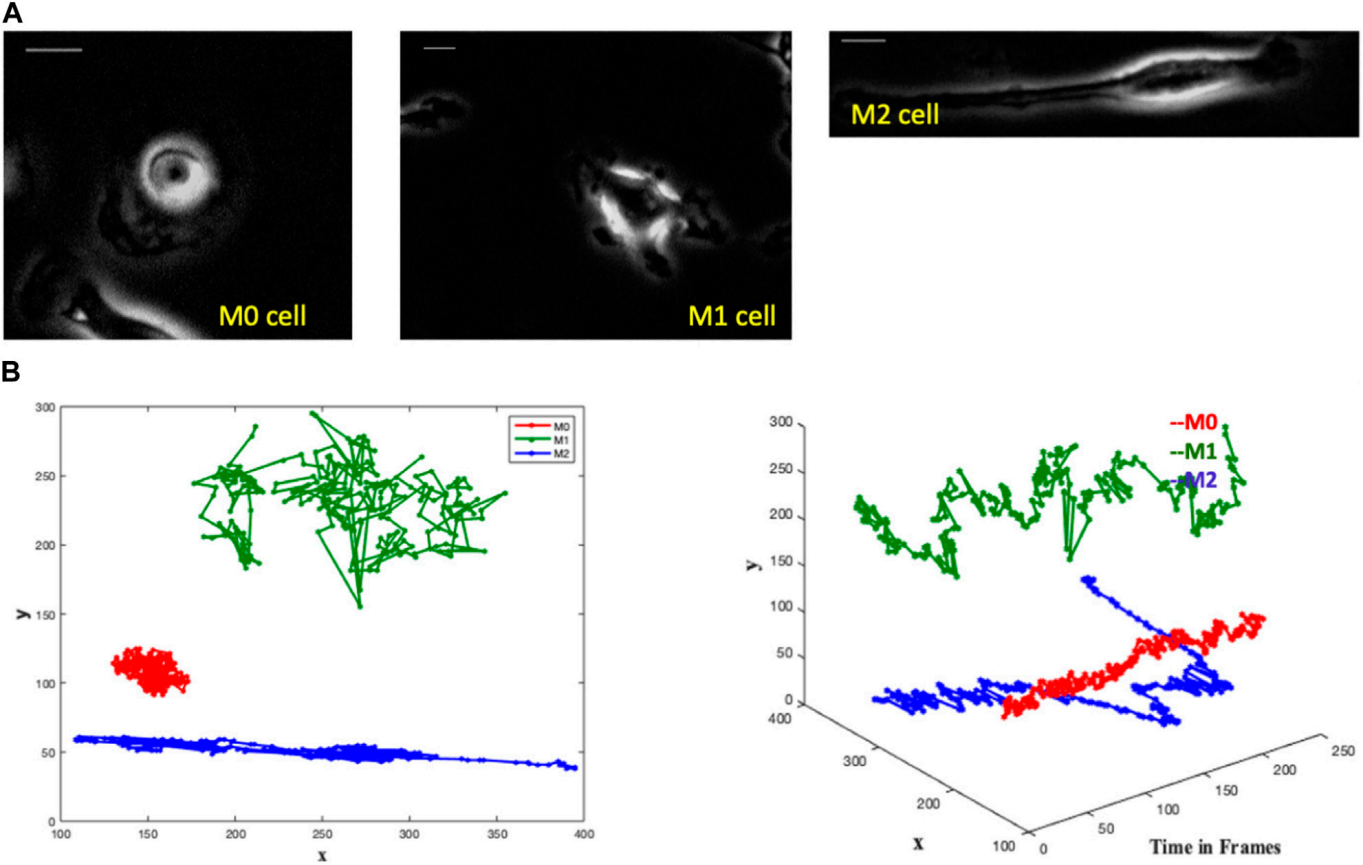
The images depict single-cell macrophages images alongside their respective trajectories, revealing distinct patterns among the three cell types (M0, M1, and M2). Trajectory analysis involves plotting the x and y positions in 2D and representing trajectories as 3D objects in (x, y, t) space. In the 3D plot, the M0 cell exhibits a spinning pattern and remains close to its initial position in the 2D plot. The M1 cell demonstrates large jumps in each frame in the 3D plot, while meandering around its initial point in the 2D plot. Conversely, the M2 cell covers a greater directional distance away from the initial point in both the 3D and 2D plots. **(A)** Single cell macrophage images of M0, M1 and M2 with an interval of 1 min/frame for 4 h - one frame from each single-cell video. Scale bar is 10 μm. **(B)** Trajectories of M0 (red), M1 (green) and M2 (blue) cells in a single graph with the comparative representation of their respective area spreads in two-dimensional and three-dimensional plots.

Each of the three videos of a single macrophage consists of time-variant phase-contrast images. A frame from each video is shown in Figure 1A. The M0 macrophage is seen as a circular cell, the M1 cell contains protrusions, and the M2 is an elongated cell. Corresponding differences in cell shape were observed in Kesapragada et al. (2024), and cell-shape-based clustering was created.

#### 2.1.1 Single macrophages trajectory analysis

We notice distinct trajectory patterns among the three cell types from the M0, M1, and M2 cell trajectories by plotting the x and y positions of their trajectories in 2D and by representing the trajectories as 3D objects in (x,y,t) space, see Figure 1B. It is seen that the M0 cell shows a spinning pattern in the 3D plot and stays close to its initial position in the 2D plot. The M1 cell makes large jumps in each frame in the 3D plot and wanders around its initial point in the 2D plot. The M2 cell moves a greater distance directionally farther away from the initial point in both 3D and 2D plots.

These variations in the cell paths are evident, suggesting the possibility of identifying cell types not only by their shape but also by their trajectory.

### 2.2 Analysis of macrophage culture images

**FIGURE 2:**
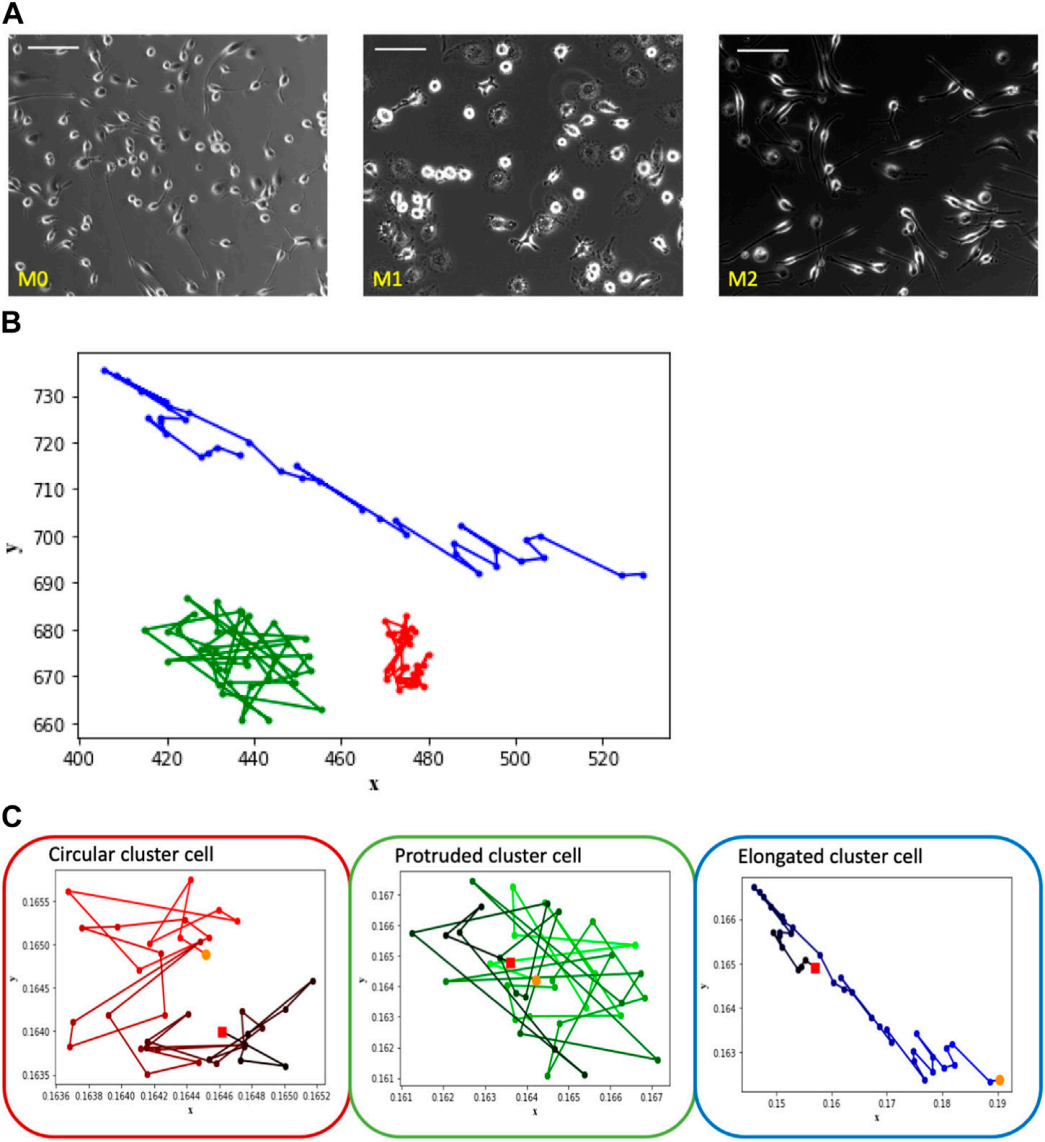
The depicted images showcase macrophage culture alongside their corresponding trajectories, categorized based on shape-based clusters. Notably, the observed patterns align with those seen in the Figure 1 images of individual cells representing M0, M1, and M2, which correspond to clusters labeled as circular, protruded, and elongated, respectively. **(A)** Macrophage images of M0, M1 and M2 cell cultures—one frame from each video set. Scale bar is 100 μm. **(B)** Trajectory patterns of the representative cells from shape Cluster C (red), Cluster P (green) and Cluster E (blue). **(C)** Trajectories of the cells shown in Figure 2B with time frame encoded by color: t = 0 corresponds to light color, t = 180 corresponds to dark. Starting point is marked as an orange dot and the ending point is marked as a red dot.

In practical settings, cell cultures typically consist of multiple cells present in each frame of recorded videos. The image sets utilized in the present study are labeled according to their culture of predominant macrophage subtypes, i.e., how cell culture was activated (M0, M1, and M2), as shown in Figure 2A. Given the macrophages’ high plasticity to transform into various functional phenotypes, it is likely that each macrophage can transform from one phenotype to another within each image set. Therefore, the challenge associated with this data set is that the labeled images may contain cells of different subtypes. For instance, M0 images may include cells of both M1 and M2 subtypes, and the same principle applies to M1 and M2 images. Therefore, we utilize the morphological clustering analysis technique described in our previous work (Kesapragada et al., 2024) to categorize cells based on their shapes. In this analysis, circular cells are assigned to Cluster C, protruded cells (cells with uneven edges) are grouped in Cluster P, and elongated cells are classified in Cluster E. Most macrophages in non-activated cell culture (M0) belong to Cluster C, M1-activated cell culture is represented mostly by Cluster P cells, and M2-activated cell culture is mostly of Cluster E, as shown in Figure 2A (see (Kesapragada et al., 2024) for details of shape-based cell clustering). In the present study, we examine the trajectory patterns of the cells of these shape clusters.

#### 2.2.1 Trajectory analysis of macrophages from their shape clusters

Consider the trajectory patterns of macrophages from the three shape clusters. The trajectories of three typical representatives of Cluster C, Cluster P, and Cluster E are shown within a single plot in Figure 2B in order to see spatial differences of cells’ trajectories. It is seen that the main difference between Cluster E cell trajectory from the trajectories of the cells of two other clusters is in the more elongated shape of the trajectory. In contrast, trajectories of the Cluster C and P cells are similar in shape but different in the size of the space occupied by the trajectory. Cluster C cell stays closer to its initial location than Cluster P cell.

However, the difference in the overall space occupied by the trajectory is not the only unique characteristic. To gain insight into temporal differences of the trajectories, consider Figure 2C that shows the trajectory patterns of these representative cells with time encoded by a color gradient from original light (t = 0) to dark color (t = 180 min). Cluster C cell exhibits a spinning pattern that occupies a smaller area, Cluster P cell demonstrates large jumps and wanders around its initial point, and Cluster E cell tends to move a greater distance in a directional manner away from the initial point. Despite variations in experiment lengths and cell densities between single and multiple-cell videos, we consistently observe similar trajectory patterns among the cells of shape clusters’ C, P, and E.

In order to quantify this behavior, we further use several mathematical measures of the trajectory pattern: convex hull perimeter and area of the trajectory, and distance measure: maximum pairwise distance between trajectory points.

#### 2.2.2 Quantitative measures of the trajectories

##### 2.2.2.1 Convex hull perimeter and area

To understand the spatial differences in Figure 2B between the cell trajectory paths of the three cell clusters, we first find the perimeter and area of the convex hull. The convex hull of a set of points is the smallest convex polygon that encloses all the points in the set (Supplementary Figure S1). The convex hull area is the total area enclosed by the convex hull, and the convex hull perimeter is the total length of the boundary that encloses the points.

**FIGURE 3:**
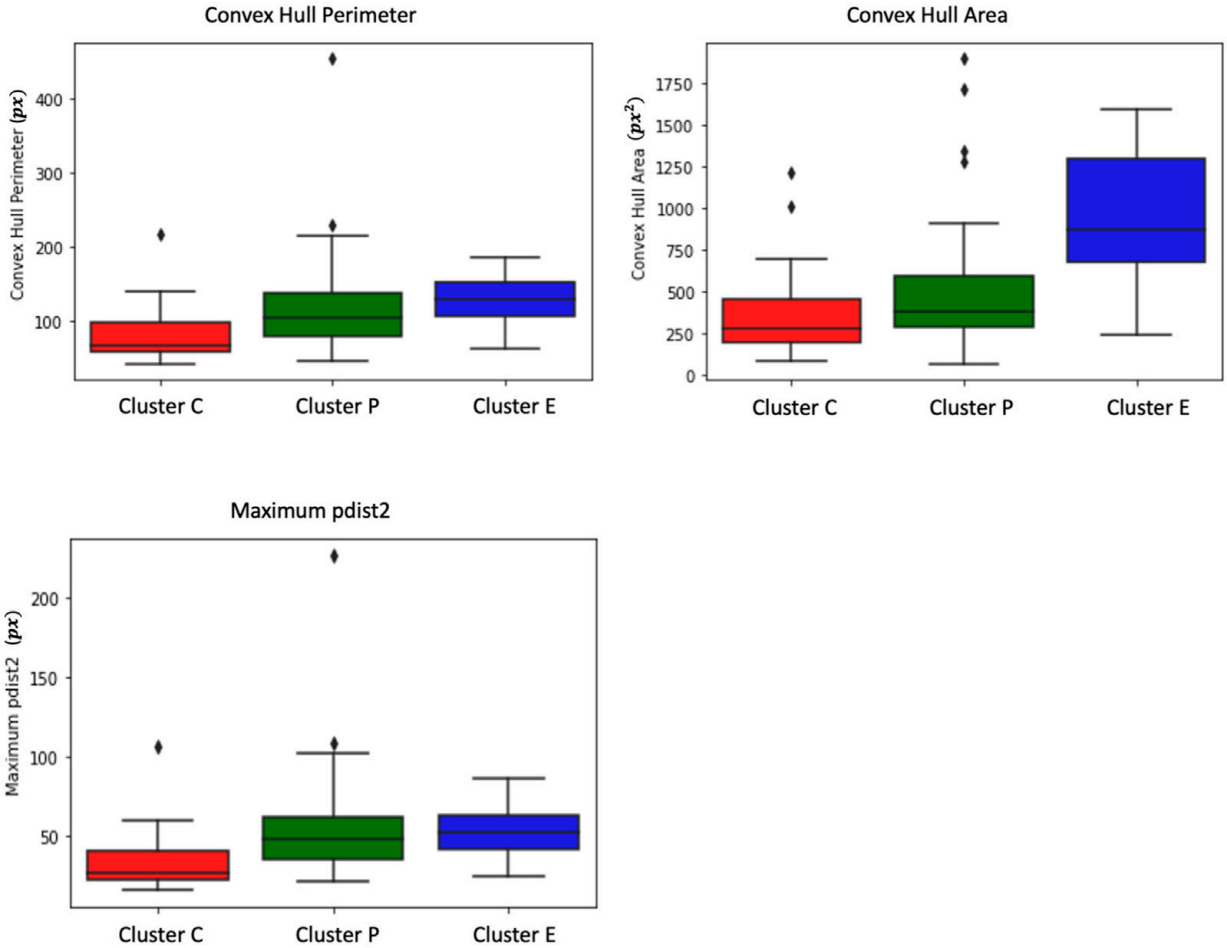
Box plots depict measures of convex hull perimeter, convex hull area, and maximum pairwise distance for trajectories from the shape-based clusters. The analysis reveals that Cluster E (elongated cells) exhibits a more substantial convex perimeter, followed by Cluster P (protruded cells) and Cluster C (circular cells). In terms of convex area, elongated cells have a larger area, followed by protruded and circular cells. While convex hull perimeter and area effectively differentiate Cluster E cells, distinguishing between Cluster P and Cluster C cells proves challenging due to the small differences in these metrics. Further examination shows that Cluster P cells display higher maximum pairwise distances compared to Cluster C cells. Interestingly, the maximum pairwise distance of Cluster E cells is similar to that of Cluster P cells.

From Figure 3, we observe that the convex perimeter of Cluster E (elongated cells) is more significant, followed by Cluster P (protruded cells) and Cluster C (circular cells). The convex area plot clearly distinguishes that the elongated cells have a larger area, followed by protruded and circular cells. Although Cluster E cells can be easily distinguished from the other two clusters of cells using convex hull perimeter and area, the difference between these metrics of Cluster P and Cluster C cells is small, and distinguishing their trajectories is challenging. Hence, we need more specific measures to distinguish the trajectories of the P and C clusters.

##### 2.2.2.2 Maximum pairwise distance

As shown in Figure 2C, Cluster P cells exhibit larger jumps between frames and show wandering behavior around the initial point. This unique characteristic of Cluster P cells can potentially be used as a distinguishing feature. To identify this characteristic, we use pairwise distance measures. We measure the pairwise distances between each pair of trajectory points and extract the maximum pairwise distance for each cell. From Figure 3, it is evident that Cluster P cells exhibit higher maximum pairwise distances than Cluster C cells. We can also observe here that the maximum pairwise distance of Cluster E cells is similar to that of Cluster P cells.

#### 2.2.3 Observations

While the quantitative features mentioned above allow us to make observations about cell migratory patterns, they are not sufficient for reliable detection of macrophage type. For example, the limitations of the convex hull, such as its sensitivity to outliers and lack of consideration for temporal ordering in trajectories, weaken its suitability as a feature of the classification model.

Having observed that macrophage trajectory patterns are specific to each cluster but still are not sufficient for automatic classification, we have developed a deep-learning model in the hope that it can have better classification performance due to capturing some trajectory features that we were unable to detect. This model gets simple characteristics of cell movement as input features and does not require the preliminary calculation of complex metrics.

## 3 Methods

### 3.1 Activation of bone marrow-derived macrophages

In each experiment, bone marrow-derived macrophages (BMDMs) were seeded into six tissue culture-treated well plates at varying densities and cultured in RPMI-1640 medium (Invitrogen) supplemented with 10% Fetal Bovine Serum (FBS) (Invitrogen) and 1× Antibiotic-Antimycotic solution (Invitrogen) overnight. For M1 activation, 100 ng/mL lipopolysaccharide (LPS) (Sigma, Cat number: L6143) was added to the culture medium, while for M2 activation, 20 ng/mL recombinant mouse interleukin-4 (IL-4) (R&D Systems, Cat number: 404-ML) was used (Ying et al., 2013). Two days post-stimulation, activated M1 and M2 macrophages were employed for morphological and motility characterizations as well as functional studies. Macrophages that did not receive any stimulation served as M0 controls.

### 3.2 Deep learning model for macrophage classification

**FIGURE 4:**
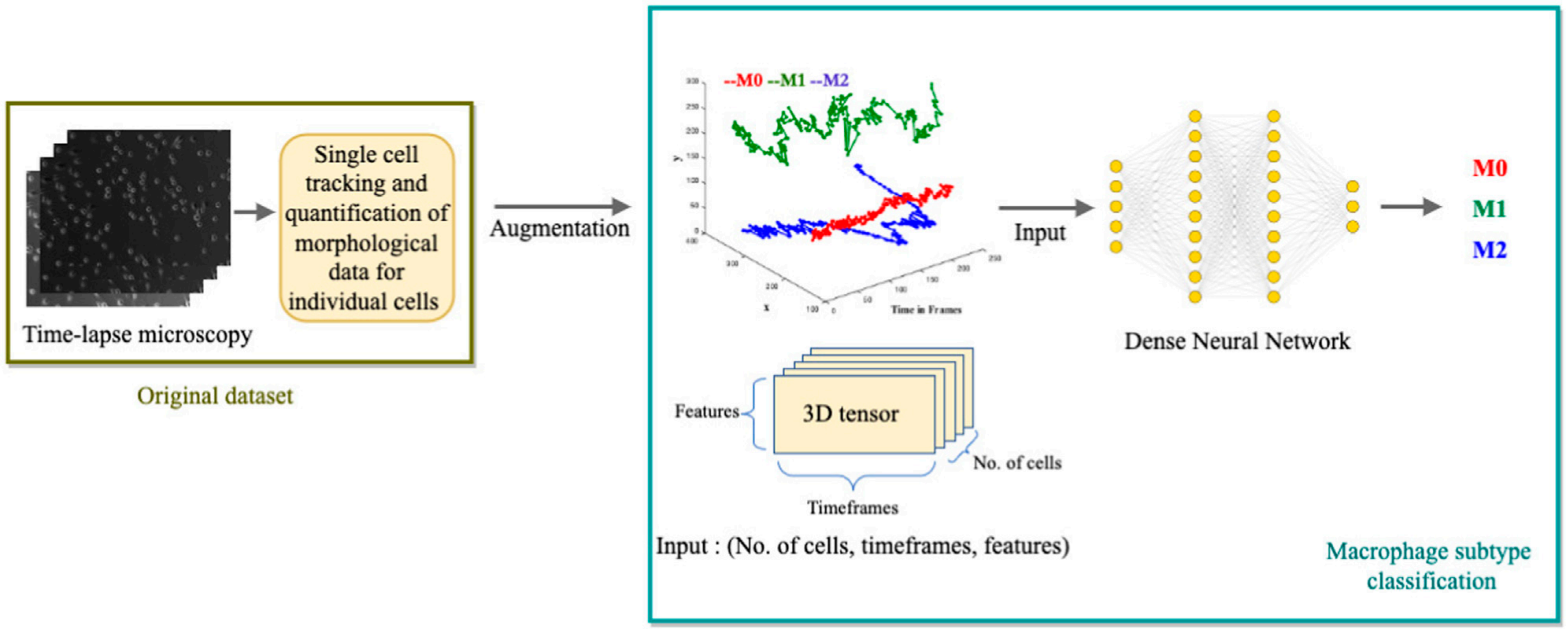
Illustration of the Deep Learning Model for macrophage subtype classification. The diagram depicts the process of extracting single-cell tracking and quantification from time-lapse images. The quantified parameters, including (x, y) positions over time, distance traveled, and cell displacement, are augmented and used as inputs for the deep learning model. The model outputs the classified macrophage subtype.

We develop a multi-class, single-label classification deep learning model where inputs are: the position coordinates (x, y) of the trajectories in time frames, the distance traveled, and the displacement of the cell. The model’s output is the classification of one of the three labels: M0, M1, or M2, assigned to their respective clusters: Cluster C (circular), cluster P (protruded), or Cluster E (elongated) (Figure 4).

#### 3.2.1 Train/test/validation data

We assign the label “M0” to cells from Cluster C, “M1” to cells from Cluster P, and “M2” to cells from Cluster E in the training data. We only include cells with a trajectory path from the first frame to the last frame, which is a total of 71 cells. We augment the data set to train the deep learning network. As the input to the model is (x, y) coordinates, we augment the data by inversion (y, x), translation (-x, y), (-x, -y), (x, -y) and inverse translation (-y, x), (-y, -x), (y, -x). These mathematical transformations generate a total of 560 cells. We shuffle the data and split 80% for training (448 cells) and 20% for validation (112 cells).

#### 3.2.2 Architecture

**FIGURE 5:**
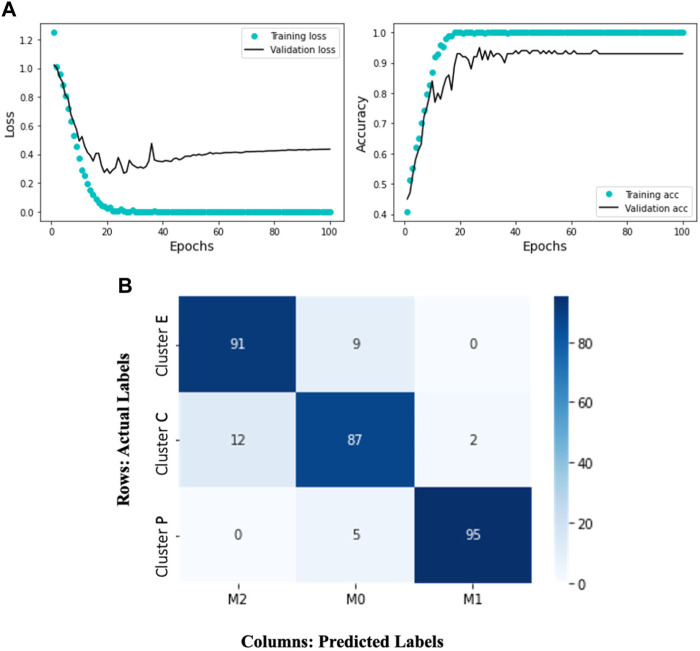
The Deep Learning model results display the training and validation loss and accuracy over 100 epochs. The accompanying heatmap confusion matrix illustrates the percentage accuracy of the validation data for the M0, M1, and M2 macrophage subtypes. In this representation, the ground truth data labels “Cluster C,” “Cluster P,” and “Cluster E” are presented in the rows, while the model-predicted labels “M0,” “M1,” and “M2” are presented in the columns. Notably, the model demonstrates 91% accuracy in predicting Cluster E (elongated) cells as M2, 95% accuracy in predicting Cluster P (protruded) cells as M1, and 87% accuracy in predicting Cluster C (circular) cells as M0. **(A)** Training and validation loss and accuracy over 100 epochs. **(B)** A heatmap confusion matrix, showing the percentage accuracy of the validation data for the M0, M1 and M2 macrophage subtypes.

For the deep learning model, we utilized Keras (Chollet, 2015), an open-source neural network library written in Python. Here, the neural networks work to separate three different classes (M0, M1, and M2). Since there are only a few labels to classify, a simple stack of eight fully connected (Dense) layers with Relu activations (Nair and Hinton, 2010) is used. The hidden units that are passed to each Dense layer are 10, 20, 32, 64, 64, 32, 20, and 10, respectively. The network’s final layer is a Dense layer with a size of 3. The network produces a 3-dimensional output vector for every input, where each dimension represents a distinct output class. The softmax activation function is used in this last layer, which generates a probability distribution over the three output classes. Categorical cross-entropy, the recommended loss function for a multi-class classification problem, is used to minimize the distance between predicted and true probability distributions. We used the RMSprop optimizer (Tieleman and Hinton, 2012) with its default learning rate to minimize the loss. The evaluation metric used is accuracy, representing the proportion of correctly classified cells. The model is trained for 100 epochs to convergence (see Figure 5A).

## 4 Model results

Given the complexity of the problem of classifying different trajectory patterns and the amount of data available, our results (Figure 5A) show that the network is not overfitting and learning effectively. We observe that the training loss decreases over time while the validation loss remains low. Similarly, the training accuracy increases while the validation accuracy remains high. This indicates that the model can generalize well to new data and is likely to perform well on new unseen data. The model’s ability to accurately classify the macrophage subtypes is supported by its successful predictions on the validation data. Figure 5B represents a confusion matrix visualization where the percentage cell counts of each class are plotted on a heatmap. In this representation, the ground truth data labels “Cluster C,” “Cluster P,” and “Cluster E” are displayed in the rows, while the model predicted labels “M0,” “M1,” and “M2” are displayed in the columns. We can observe that the model has an accuracy of 91% in predicting Cluster E (elongated) cells as M2, 95% accuracy in predicting Cluster P (protruded) cells as M1, and 87% accuracy in predicting Cluster C (circular) cells as M0. We can see that the model has identified 12% of the Cluster C cells as M2 and 2% as M1. It is important to note that identifying M0 cells can be challenging, as they may exist in a continuum or may continuously transform into other subtypes. This trained model is applied to test single-cell macrophage images (Figure 1A), which accurately categorized the cells into M0, M1, and M2.

## 5 Discussion

Cell-trajectory-based and cell-shape-based methods can potentially classify macrophage subtypes more accurately at the single-cell level. This differs from other methods, such as q-PCR, that give information about cell population subtypes.

As we observed in the cell morphology analysis (Kesapragada et al., 2024), each cell culture (M0, M1, or M2) is dominantly represented by a corresponding morphology cluster: M0 cells are circular, M1 cells are protruded, M2 cells are elongated, but the shape-based clusters were nonetheless diluted.

Current analysis of macrophage trajectories revealed typical cell movement patterns corresponding to cell shape clusters: circular cells spin at their initial position, protruded cells wander, and elongated cells move in a specific direction. However, trajectory-based clusters do not exactly correspond to shape-based clusters. This implies a variation in the resulting cell phenotype of macrophage culture activated into M1, M2, or non-activated (M0).

We also note that it is challenging to arrive at a set of metrics that holistically capture all the shape and movement features of the cell.

Several image-based models for macrophage type identification have been published previously. In Pavillon et al. (2018), the authors created a model for noninvasive distinguishing between cell types employing activated and non-activated macrophages for testing. The classification model was linear, allowing greater biological interpretation. However, it required multiple sources of information as input, including phase microscopy images, Raman spectra, and autofluorescence microscopy. In Rostam et al. (2017), the authors used image-based machine learning approach to classify M1, M2, naive macrophages, and monocytes. Their algorithm relied on various cell metrics, including details related to the nucleus and cytoskeleton, and requires high-quality images.

Our deep learning model relies on the raw cell trajectory data in the x-y plane, as well as the cell displacement and traveling distance. It automatically captures the features of cell motion unseen by the human eye and demonstrates good accuracy.

## 6 Conclusion

Our study revealed unique migratory patterns and distinct morphology in the three subtypes of macrophages. M0, M1, and M2. By analyzing their trajectories and computing various quantitative measures, such as perimeter, area of the convex hull, and pairwise distances, we observed clear differences in the migratory patterns of these macrophage cell types. However, building a classification model with predetermined features is challenging in this context.

Therefore, we take a different approach by developing a deep learning model that incorporates the trajectory path and shape of cells, which proves to be more effective in accurately classifying macrophage subtypes. The correlation between cell shape and trajectory patterns can be highly valuable in future scenarios where obtaining precise cell morphology data is challenging. Additionally, identifying cells based on their migration patterns through phase-contrast microscopy has the potential to eliminate the requirement for high-quality imaging and provide more reliable methods for analyzing blurry images.

## Data Availability

The datasets presented in this study can be found in online repositories. The names of the repository/repositories and accession number(s) can be found in the article/Supplementary Material.
