# Supplementary material for: Deep learning classification for macrophage subtypes through cell migratory pattern analysis
Source: Front Cell Dev Biol. 2024 Feb 7;12:1259037. doi: 10.3389/fcell.2024.1259037 (PMC10879298; doi:10.3389/fcell.2024.1259037)
Supplement: Supplementary file 1 [file DataSheet1.pdf]

Supplementary Material

1 SUPPLEMENTARY DATA

The code and the datasets to replicate the results for this study can be found on github: https://github.com/Gomez-Lab/Macrophages_TrajectoryPatternAnalysis.

2 SUPPLEMENTARY FIGURES

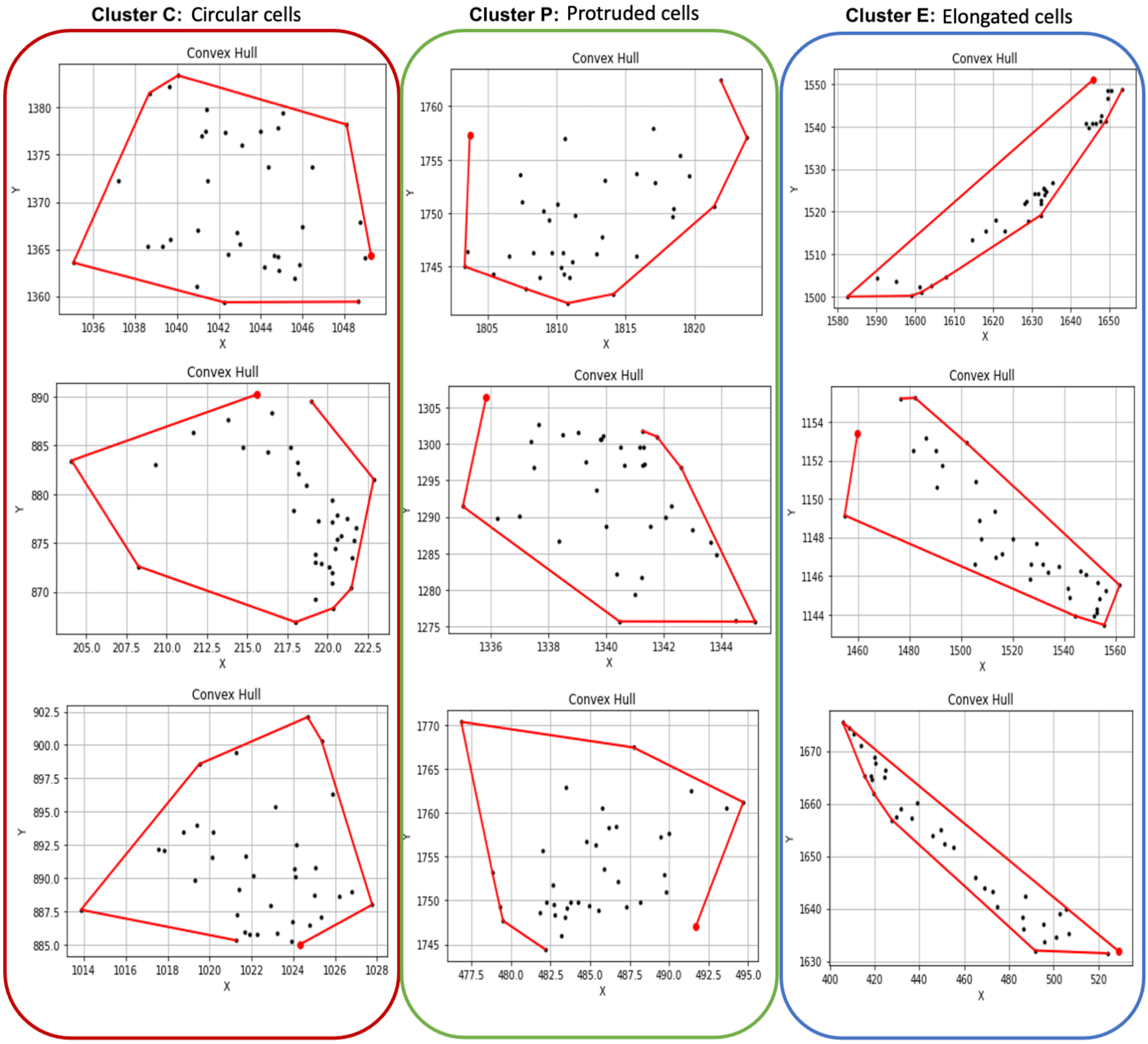

Figure S1. The red boundary in each plot represents the convex hull of a set of points, denoting the smallest convex polygon that encloses all the points in the set. These plots correspond to cells in Cluster C, P, and E, representing circular, protruded, and elongated cell types, respectively.
